# Spiked Helmet Sign in the Early Post‐Heart Transplantation Period: A Dramatic STEMI Mimic in a Structurally Normal Donor Heart

**DOI:** 10.1111/anec.70243

**Published:** 2026-10-20

**Authors:** Drizia Renally Macedo Lima, Gutemberg de Sousa Dantas Segundo, Gabriel Pelegrineti Targueta, Roberta Tavares Barreto Teixeira, Alex Tiburtino Meira, Guilherme Augusto Teodoro Athayde

**Affiliations:** ^1^ Federal University of Paraíba (UFPB) João Pessoa Paraíba Brazil; ^2^ Dom José Maria Pires Metropolitan Hospital João Pessoa Paraíba Brazil

**Keywords:** electrocardiography, heart transplantation, myocardial infarction, spiked helmet sign

## Abstract

A 36‐year‐old woman with severe dilated cardiomyopathy (LVEF 14%, NYHA IV) underwent heart transplantation. Postoperatively, she developed subarachnoid hemorrhage and septic shock. The ECG revealed a spiked helmet sign (SHS) pattern, with repolarization abnormalities and QT prolongation. Despite transient clinical improvement and resolution of the ECG changes after intensive support, the patient died due to infectious complications. This case highlights the occurrence of SHS in the early posttransplant period in a structurally normal donor heart, a previously unreported presentation, reinforcing its extracardiac origin and association with critical illness and poor prognosis.

## Graphical abstract

A 36‐year‐old woman developed Spiked Helmet Sign after heart transplantation complicated by subarachnoid hemorrhage and septic shock. Although the ECG changes resolved, she died from infectious complications, highlighting SHS as a marker of critical illness and poor prognosis.

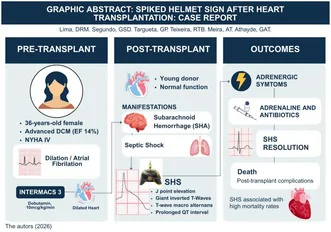

## 1 Case Presentation

A 36‐year‐old woman with advanced dilated cardiomyopathy, left ventricular ejection fraction 14%, New York Heart Association (NYHA) functional Class IV, refractory to medical therapy and multiple hospitalizations at a University Hospital due to decompensated heart failure, underwent heart transplantation at a referral center.

Preoperatively, the baseline electrocardiogram (ECG) showed atrial fibrillation, with signs of left ventricular hypertrophy. At admission, she was classified as SCAI (Society for Cardiovascular Angiography and Intervention) stage C and was hemodynamically stable on dobutamine at 10 μg/kg/min.

The transplantation was uneventful, with a cardiopulmonary bypass time of 2 h. The donor organ was obtained from a young individual with brain death following traumatic brain injury and showed no structural abnormalities, regional wall motion abnormalities, or ventricular dysfunction, as demonstrated by preoperative echocardiogram.

In the immediate postoperative period, the ECG showed sinus rhythm with a right bundle branch conduction disturbance and a QRS duration < 120 ms (Figure 1A). On postoperative Day 2, her neurological status deteriorated due to a subarachnoid hemorrhage (SAH) involving the left frontal lobe (Figure 1C). On postoperative Day 12, the patient developed septic shock of pulmonary origin, requiring escalating doses of vasopressors (norepinephrine and vasopressin) and inotropic support (dobutamine) to achieve hemodynamic stabilization.

**FIGURE 1:**
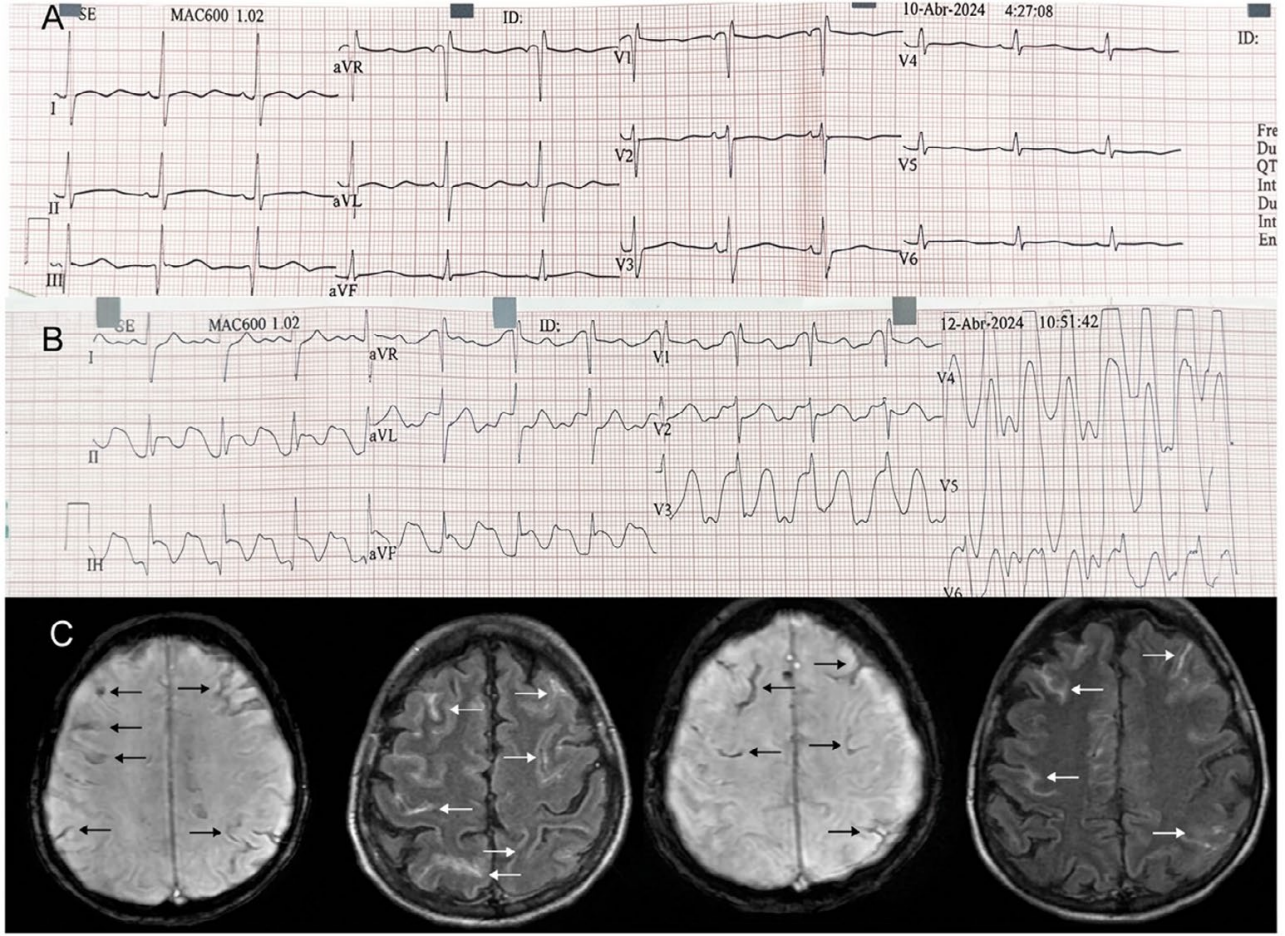
Electrocardiogram and brain MRI demonstrating spiked helmet sign and subarachnoid hemorrhage. (A) Posttransplant ECG showing sinus rhythm, right bundle branch conduction delay, and QRS < 120 ms. (B) Concomitant with the worsening of the clinical and hemodynamic status, the patient's ECG shows a rapid‐activation QRS, downsloping J‐point elevation, and QT interval prolongation with ventricular repolarization abnormalities, presenting with a giant negative T‐wave extending over the subsequent QRS. Additionally, T‐wave macro‐alternans is observed, a pattern suggestive of the Spiked Helmet Sign. (C) Subarachnoid hemorrhage demonstrated on MRI. The two images on the left correspond to susceptibility‐weighted sequences, showing signal hypointensity, whereas the two images on the right are FLAIR images demonstrating hyperintense signal. *Source:* The authors (2026).

At this point, the ECG demonstrated rapid QRS activation and descending J‐point elevation, predominantly in the precordial leads (V3–V6), with associated abnormalities in the inferior leads (II, III, and aVF) and aVL. The QT interval was prolonged, with abnormal ventricular repolarization characterized by a giant negative T wave extending into the subsequent QRS complex, in addition to T‐wave macroalternans, a pattern suggestive of the spiked helmet sign (SHS) (Figure 1B). The cardiac troponin level was 9.74 ng/mL on the first day and 9.96 ng/mL after 24 h, remaining persistently elevated thereafter without significant variation.

After the addition of epinephrine and the onset of antibiotic effect, the patient showed hemodynamic improvement, stabilization of parameters, and resolution of the SHS pattern. Myocardial function remained normal on subsequent echocardiograms. However, after 49 days of hospitalization, she died from sepsis in the setting of post‐heart transplantation immunosuppression.

## 2 Discussion

We report a case of SHS occurring in the early postoperative period after heart transplantation, in a recipient of a young and structurally normal donor heart, associated with hemodynamic instability and subarachnoid hemorrhage. To our knowledge, this presentation has not been previously reported. This case highlights the extracardiac origin of this rare electrocardiographic finding and reinforces its association with a dismal prognosis.

The SHS is a rare electrocardiographic pattern characterized by a baseline elevation encompassing the QRS complex, resembling a German military helmet (Littmann 2019). Although its appearance mimics acute myocardial infarction (ST‐elevation myocardial infarction [STEMI]), SHS is generally associated with critical noncardiac conditions (Littmann 2019; Wang et al. 2023).

The differential diagnosis between SHS and STEMI can be aided by serial measurements of cardiac biomarkers, as SHS does not typically cause a progressive rise in troponin levels (Littmann and Monroe 2011). Studies suggest that this sign has been associated with mortality rates of up to 75% (Wang et al. 2023).

First described in 2011, various complications have been associated with the presence of SHS, including SHA, pneumothorax, severe pneumonia, ischemia, intestinal obstruction, and autonomic dysfunction (Takotsubo cardiomyopathy, spinal cord injury, cocaine abuse, and stellate ganglionectomy) (Wang et al. 2023).

In our case, SAH and septic shock triggered intense systemic stress. Unlike most cases, the patient had a structurally normal transplanted heart, with no evidence of a primary cardiac trigger. This reinforces the hypothesis that SHS reflects a neurocardiogenic and extracardiac process rather than a primary cardiac event.

Moreover, the resolution of the SHS pattern with hemodynamic stabilization suggests a functional and reversible origin. The association of a transplanted heart, severe neurological injury, and systemic inflammatory response highlights the extracardiac nature of the phenomenon, reinforcing SHS as a marker of critical illness and poor prognosis, even in the absence of structural heart disease.

The topographic distribution of SHS on the electrocardiogram often correlates with the underlying etiology: its presence in the inferior leads (II, III, and aVF) suggests acute abdominal events; involvement of the precordial leads suggests thoracic conditions; and diffuse or generalized patterns are typically associated with metabolic or neurological disorders (Wang et al. 2023). In this clinical setting, predominantly neurological and metabolic etiologies are consistent with the diffuse and nonspecific electrocardiographic patterns described in critically ill patients (Figure 1B).

The pathophysiology of SHS has not yet been fully elucidated. A mechanical theory attributes the sign to voltage artifacts generated by the diaphragm in the setting of increased thoracoabdominal pressure; whereas an electrophysiological theory proposes that it results from the superimposition of prolonged ventricular repolarization (T‐U wave from the previous cycle) onto the subsequent QRS complex, a process mediated by intense adrenergic stress and QT interval prolongation (Littmann and Monroe 2011).

In the present case, SHS is not typically of primary ischemic origin and is instead associated with critical noncardiac conditions. The literature links this electrocardiographic pattern to an adrenergic surge and increased thoracoabdominal or intracranial pressure, findings that are consistent with the patient's postoperative course complicated by SAH and septic shock.

Pathophysiologically (Littmann and Monroe 2011), this case supports an electrophysiological mechanism. The use of high‐dose inotropes (dobutamine and epinephrine) and the acute neurological insult resulted in QT interval prolongation and marked abnormalities in ventricular repolarization. This led to superimposition of the preceding T‐U wave onto the subsequent QRS complex, producing the characteristic SHS morphology, defined by a baseline elevation preceding the QRS complex.

T‐wave alternans observed on the patient's ECG is a recognized marker of myocardial electrical instability under severe adrenergic stress, suggesting heightened susceptibility of the transplanted myocardium to systemic catecholaminergic stimulation. In addition, the distribution of ECG changes and the absence of a progressive rise in troponin levels, as typically seen in acute myocardial infarction, further support the differential diagnosis described in the literature (Littmann and Monroe 2011).

This case report contributes novel findings to the literature by describing a patient with SHS after heart transplantation, complicated by subarachnoid hemorrhage and septic shock. The patient's young age (36 years) is also a rare clinical feature, as studies report a mean age of 61 years in these patients (Wang et al. 2023).

The present case reinforces the importance of recognizing SHS as an electrocardiographic marker of severity, associated with a poor prognosis and high mortality rate. In the post–heart transplantation setting, the manifestation of SHS highlights the challenge of differentiating it from acute myocardial infarction. In this context, early recognition is crucial to avoid invasive diagnostic interventions, such as unnecessary cardiac catheterizations, and to direct attention to the true underlying cause.

## Author Contributions

**Drizia Renally Macedo Lima:** literature review, writing – original draft, writing – review and editing. **Gutemberg de Sousa Dantas Segundo:** literature review, writing – original draft, writing – review and editing. **Gabriel Pelegrineti Targueta:** conceptualization, data collection. **Roberta Tavares Barreto Teixeira:** conceptualization, data collection. **Alex Tiburtino Meira:** supervision, writing – review and editing. **Guilherme Augusto Teodoro Athayde:** conceptualization, data collection, formal analysis, supervision, writing review and editing. All authors have read and agreed to the published version of the manuscript and satisfy all four ICMJE authorship criteria.

## Funding

The authors have nothing to report.

## Conflicts of Interest

The authors declare no conflicts of interest.

## Data Availability

The data that support the findings of this study are available on request from the corresponding author. The data are not publicly available due to privacy or ethical restrictions.
